# Handgrip strength assessment at baseline in addition to bone parameters could potentially predict the risk of curve progression in adolescent idiopathic scoliosis

**DOI:** 10.3389/fped.2023.1258454

**Published:** 2023-11-03

**Authors:** Rufina Wing Lum Lau, Ka Yee Cheuk, Vivian Wing Yin Hung, Fiona Wai Ping Yu, Elisa Man Shan Tam, Lyn Lee Ning Wong, Jiajun Zhang, Wayne Yuk Wai Lee, Jack Chun Yiu Cheng, Tsz Ping Lam, Adam Yiu Chung Lau

**Affiliations:** ^1^ School of Medical and Health Sciences, Tung Wah College, Hong Kong, Hong Kong SAR, China; ^2^ Department of Orthopaedics and Traumatology, The Chinese University of Hong Kong, Hong Kong, Hong Kong SAR, China; ^3^ SH Ho Scoliosis Research Laboratory, Faculty of Medicine, The Chinese University of Hong Kong, Hong Kong, Hong Kong SAR, China; ^4^ Joint Scoliosis Research Center of the Chinese University of Hong Kong and Nanjing University, The Chinese University of Hong Kong, Hong Kong, Hong Kong SAR, China; ^5^ Bone Quality and Health Centre, Department of Orthopaedics and Traumatology, The Chinese University of Hong Kong, Hong Kong, Hong Kong SAR, China

**Keywords:** adolescent idiopathic scoliosis, handgrip, strength, bone, curve progression

## Abstract

**Introduction:**

Adolescent idiopathic scoliosis (AIS) is characterized by deranged bone and muscle qualities, which are important prognostic factors for curve progression. This retrospective case–control study aims to investigate whether the baseline muscle parameters, in addition to the bone parameters, could predict curve progression in AIS.

**Methods:**

The study included a cohort of 126 female patients diagnosed with AIS who were between the ages of 12 and 14 years old at their initial clinical visit. These patients were longitudinally followed up every 6 months (average 4.08 years) until they reached skeletal maturity. The records of these patients were thoroughly reviewed as part of the study. The participants were categorized into two sub-groups: the progressive AIS group (increase in Cobb angle of ≥6°) and the stable AIS group (increase in Cobb angle <6°). Clinical and radiological assessments were conducted on each group.

**Results:**

Cobb angle increase of ≥6° was observed in 44 AIS patients (34.9%) prior to skeletal maturity. A progressive AIS was associated with decreased skeletal maturity and weight, lower trunk lean mass (5.7%, *p* = 0.027) and arm lean mass (8.9%, *p* < 0.050), weaker dominant handgrip strength (8.8%, *p* = 0.027), deranged cortical compartment [lower volumetric bone mineral density (vBMD) by 6.5%, *p* = 0.002], and lower bone mechanical properties [stiffness and estimated failure load lowered by 13.2% (*p* = 0.005) and 12.5% (*p* = 0.004)]. The best cut-off threshold of maximum dominant handgrip strength is 19.75 kg for distinguishing progressive AIS from stable AIS (75% sensitivity and 52.4% specificity, *p* = 0.011).

**Discussion:**

Patients with progressive AIS had poorer muscle and bone parameters than patients with stable AIS. The implementation of a cut-off threshold in the baseline dominant handgrip strength could potentially be used as an additional predictor, in addition to bone parameters, for identifying individuals with AIS who are at higher risk of experiencing curve progression.

## 1. Introduction

Adolescent idiopathic scoliosis (AIS) is a complex three-dimensional spinal deformity (1). AIS is a multifactorial disease, and its etiology and pathogenesis are not fully understood (1). Systemic low areal bone mineral density (aBMD) in 30% of AIS girls was identified using dual energy x-ray absorptiometry (DXA) (2). Over 80% of AIS patients with low BMD at baseline are associated with persistent osteopenia in their early adulthood (3). Recent studies utilizing high-resolution peripheral quantitative computed tomography (HR-pQCT) to assess the true volumetric bone mineral density (vBMD) and micro-architecture of bone revealed that individuals with AIS exhibited deranged bone qualities including bone macro- and microstructure and lower bone mechanical properties at the distal radius when compared with normal female adolescents (4, 5). It is speculated that systemic low bone mass tends to increase the chance of vertebral bone wedging and contributes to the increase of spinal curvature (6). Low aBMD at the femoral neck (7) and cortical vBMD at the distal radius (8) were identified to be important and independent prognostic factors for curve progression in AIS. However, the underlying cause of systemic low bone mass in AIS is not fully understood. Emerging evidence showed that AIS patients presented abnormal bone turnover and mineralization (9, 10).

Abnormal muscle qualities such as lower skeletal muscle mass were also observed in AIS patients (11). AIS girls with a right thoracic curve had higher T2-weighted signal intensity in magnetic resonance imaging (MRI) and a greater fatty component in the multifidus muscle on the concave side when compared with the age-matched controls without scoliosis (12). The muscle volume was also larger in the convex side, while the concave side displayed higher levels of endomysial and perimysial fibrosis and fatty infiltration (13, 14). The electromyographic activity at the convex side exhibited an increase, accompanied by more type I muscle fiber, which was positively correlated with the curve severity in AIS patients (15).

The bone and muscle closely interact with each other via both biochemical and mechanical pathways (16). The skeletal growth is regulated and modulated by mechanical loading through a mechanotransduction process that involves paracrine and endocrine signaling (17). The muscle serves as the primary source of mechanical stimuli necessary for the bone to develop its strength and mass. From a mechanical perspective, the accelerated skeletal growth with lower bone density observed in AIS patients may indicate that they probably experience less mechanical loading compared with healthy controls due to reduced muscle mass and strength (18). The differential growth of bone and muscle tissues could be a possible mechanism contributing to the development and progression of AIS (18). The association between muscle and bone qualities has been investigated in normal children (19), elderly (20, 21), and patients with type 2 diabetes mellitus (22), but this is poorly understood among patients with AIS. To the best of our knowledge, no study focused on the association between bone qualities, muscle mass and strength, and curve progression in AIS patients. This retrospective case–control study aims to investigate whether baseline muscle and bone parameters in AIS could predict curve progression.

## 2. Materials and methods

### 2.1. Subjects

The medical records of a scoliosis clinic in a local hospital were retrieved and reviewed between the period 2019 and 2022. The study included 126 subjects who met the following inclusion criteria: (1) female aged between 12 and 14 when first diagnosed with AIS, which was confirmed through clinical examination and standard standing posteroanterior radiograph of the entire spine (23), (2) available data on baseline hand grip strength and bone parameters, (3) without prior treatment such as brace and physiotherapeutic scoliosis specific exercises at their first visit, and (4) with longitudinal follow-up every 6 months and reaching skeletal maturity (defined as age ≥16 years old and year since menarche of ≥2) before 2018. All subjects exhibited an average maximum Cobb angle of 21.7° ± 6.64° at the first clinical visit. The median of follow-up duration was 4.08 years. The exclusion criteria included Cobb angle of <10° or ≥40° at first visit, scoliosis with any known etiology such as congenital scoliosis, neuromuscular scoliosis, scoliosis of metabolic etiology, scoliosis with skeletal dysplasia, known endocrine and connective tissue abnormalities, known heart condition or other diseases that could affect the safety of exercise, eating disorders or gastrointestinal malabsorption disorders, and currently taking medication that affects bone or muscle metabolism. The subjects were categorized into two groups, namely the progressive AIS (increase in Cobb angle ≥6°) group and the stable AIS (increase in Cobb angle <6°) group, according to the criteria established by the Scoliosis Research Society (SRS). All procedures performed in studies involving human participants were in accordance with the ethical standards of The Joint Chinese University of Hong Kong—New Territories East Cluster Clinical Research Ethics Committee (reference no.: CREC-2009.491 and CREC-2009.020) and with the 1964 Helsinki declaration and its later amendments or comparable ethical standards. Written informed consent was obtained from all subjects included in this study and their guardians before undertaking the evaluations.

### 2.2. Handgrip strength

Handgrip strength was measured using the standard dynamometer (T.K.K. 5401 Grip-D; Takei Scientific Instruments Co., Ltd., Japan) during the first visit prior to any intervention. The subjects were instructed to hold the dynamometer and position their arm aligning with the trunk, pointing downward. Three trials to evaluate maximal isometric contraction in both hands were measured (24). The highest value at each hand was used for analysis. Previous studies have indicated a high level of reliability and validity of handgrip strength result among untrained adolescents (25).

### 2.3. Arm lean mass

The body composition was evaluated using a standardized protocol by bioelectric impedance analysis (BIA) (InBody 720, Biospace, Korea), which utilized an eight-polar bioimpedance method using a multi-frequency current (11). The skeletal muscle mass was computed based on the muscle mass of the limbs, which predominantly consists of skeletal muscle and accounts for 70% of the total skeletal muscle mass in the body. The estimated whole body, arm, and leg lean mass were reported.

### 2.4. Bone qualities and bone mechanical properties of the distal radius

The bone parameters of the non-dominant distal radius of the subjects were measured using HR-pQCT (XtremeCT I; Scanco Medical AG, Switzerland). A fixed offset at 5 mm proximal to the most proximal limit of the inner aspect of epiphyseal growth plate of the radius was used to define the region of interests (5). A total of 110 slices were acquired, with a voxel size of 82 µm^3^ and a thickness of 9.02 mm. An image that exhibited severe motion artifact was excluded from the analysis. The periosteal surface of the radius was contoured by a single technician to minimize the inter-operator variation. A Gaussian filter was used to remove the noise signal (26). An automated threshold-based algorithm was used to segment the cortical bone and trabecular bone (26). The total area, cortical area, and trabecular area of the bone were measured. The cortical thickness was calculated as the mean cortical volume divided by the outer cortical surface area (27). The total, cortical, and trabecular vBMD were calculated in whole bone envelope, cortical bone, and trabecular bone, respectively.

Bone mechanical properties, including stiffness and estimated failure load, were calculated by finite element analysis using the software provided by the manufacturer (µFEA Solver v.1.15; Scanco Medical, Switzerland). The finite element model contained eight-node brick elements with a volume of 82 µm^3^ and assumed that bone tissue is an isotropic and elastic material characterized by a Young's modulus of 10 GPa and a Poisson's ratio of 0.3 (28). A uniaxial compression test was conducted, applying a 1% strain along the axial direction. The estimated failure load was determined when the effective strain of the 1 mm^3^ elements in the model exceeded 7,000 microstrain (29).

### 2.5. Areal bone mineral density of the femoral neck

aBMD of the non-dominant femoral neck (g/cm^2^) was measured using dual energy x-ray absorptiometry (DXA, XR-46; Norland Medical Systems, Fort Atkinson, WI, USA). The details of DXA measurement was presented in a study conducted by Cheng et al. (30). The z-score was calculated with reference to a normative dataset consisting of local ethnic Chinese girls.

### 2.6. Anthropometric and maturity assessment

Body weight and arm span were measured. Given that AIS patients had a reduction of body height due to spinal deformity, measuring of arm span was used as a means of calculating body mass index (BMI) (BMI by arm span = body weight/arm span^2^). The self-reported age of menarche corrected to the nearest month was recorded. Skeletal age was evaluated using the Thumb Ossification Composite Index (TOCI) (range 1–8) on a left hand radiograph. TOCI is a validated staging system reflecting the ossification pattern of the thumb epiphyses and the adductor sesamoid bone with high accuracy for predicting skeletal maturity and comparable with the Sanders simplified maturity system (31). A longitudinal study has shown that peak height velocity in AIS girls can be predicted by TOCI, and majority attained their peak height velocity at TOCI stage 5 (31).

### 2.7. Dietary calcium intake and physical activity level

The subjects were required to provide the details regarding their usual consumption habits and frequency in the past 1 year using a modified version of the Chinese Food Frequency Questionnaire for evaluating the dietary calcium (Ca) intake. The assessment of dietary nutrient intake was conducted using the Food Processor Nutrition Analysis and Fitness software version 7.9 (Esha Research, Salem, OR, USA), and the composition of some local foods was determined based on the China Food Composition Table (Institute of Nutrition and Food Safety, 2002) (32). The physical activity level of the past 1 year was assessed using the Chinese version of the Modified Baecke Questionnaire (33).

## 3. Statistical analysis

Data were tested using the Kolmogorov–Smirnov test for normality, and normally distributed data were presented as mean ± SD. Student's *t*-test was used to compare the difference on parameters between the progressive and stable AIS groups. Skewed data was presented as medians (range), and Mann–Whitney *U* test was used for comparison between groups. Body weight was not included as the covariates in the regression model since it was highly correlated with the lean mass (*r* = 0.891 and 0.931 in AIS and the controls, respectively). SPSS statistic software (version 24; SPSS Inc., Chicago, IL, USA) was used for statistical analyses. The analyses were two-tailed. *P*-values <0.05 were considered statistically significant.

## 4. Results

All patients with AIS were clinically followed up until they reach skeletal maturity. A total of 44 AIS patients (34.9%) had curve progression with a change of Cobb angle ≥6° prior to skeletal maturity. At baseline, it was observed that progressive AIS patients had similar age, curve severity, and lifestyle parameters, but lower weight and TOCI as compared with stable AIS. The initial maximum Cobb angle and Cobb angle at maturity were similar between the progressive and stable AIS groups at baseline (22.5 ± 7.5 vs. 21.3 ± 6.1, *p* = 0.309), but both angles were significantly higher at maturity for the progressive AIS group (32.8 ± 11.5 vs. 20.9 ± 6.5, *p* < 0.001). For muscle parameters, the progressive AIS group had lower trunk (5.7%, *p* = 0.027) and arm lean mass at both the dominant (9.2%, *p* = 0.035) and non-dominant (8.6%, *p* = 0.041) sides, and a weaker handgrip strength at the dominant side (8.8%, *p* = 0.027). For bone parameters, the progressive AIS group showed a reduced femoral neck aBMD (6.3%, *p* = 0.007), deranged cortical compartment [29.1% smaller area (*p* = 0.003) and 6.5% lower vBMD (*p* = 0.002)], and lower bone mechanical properties [13.2% lower stiffness (*p* = 0.005) and 12.5% lower estimated failure load (*p* = 0.004)] when compared with the stable AIS group. More patients with progressive AIS (75%) when compared with patients with stable AIS (36.6%) received either bracing or surgical intervention between baseline and until skeletal maturity. Basic characteristics, lifestyle, and muscle and bone parameters in progressive and stable AIS are shown in Table 1.

**Table 1:** Basic characteristics, lifestyle, and muscle and bone parameters in individuals with progressive AIS and stable AIS.

	Progressive (*N* = 44)	Stable (*N* = 82)	*p*-value
Age (year)^a^	12.97 (12.26–13.53)	13.04 (12.69–13.52)	0.295
Initial maximum Cobb angle^b^	22.5 ± 7.5	21.3 ± 6.1	0.309
Cobb angle at maturity^b^	32.8 ± 11.5	20.9 ± 6.5	<0.001*
Received bracing/ surgery	75.0%	36.6%	<0.001*
Anthropometric parameters			
Weight (kg)^a^	40.2 (36.1–43.3)	42.4 (38.93–49.2)	0.010*
Maturity			
TOCI^a^	7 (5, 8)	7 (6, 8)	0.012*
Menarche age (year)^a^	12.24 (11.53–12.8)	12.02 (11.27–12.7)	0.486
Onset of menarche^c^	61.4%	75.6%	0.094
Lifestyle parameters			
Physical activity level^a^	7.20 (6.53–7.79)	7.29 (6.63–7.83)	0.651
Dietary Ca intake (mg/day)^a^	586.02 (354.76–846.65)	594.24 (433.13–826.91)	0.472
Muscle parameters			
Muscle mass by BIA			
Skeletal muscle mass (kg)^b^	16.74 ± 2.25	17.61 ± 2.45	0.053
Trunk lean mass (kg).	13.08 ± 1.8	13.87 ± 1.91	0.027*
Non-dominant arm lean mass (kg)^b^	1.17 ± 0.27	1.28 ± 0.29	0.041*
Dominant arm lean mass (kg)^b^	1.19 ± 0.27	1.31 ± 0.3	0.035*
Non-dominant leg lean mass (kg)^b^	4.65 ± 0.75	4.94 ± 0.8	0.052
Dominant leg lean mass (kg)^b^	4.66 ± 0.74	4.94 ± 0.8	0.058
Maximum handgrip strength			
Non-dominant (kg)^b^	17.14 ± 4.27	18.65 ± 4.48	0.068
Dominant (kg)^a^	18.25 (15.25–19.88)	20 (16.5–23)	0.027*
Bone parameters (non-dominant)			
DXA parameter			
Femoral neck aBMD (g/cm^2^)^b^	0.705 ± 0.082	0.752 ± 0.106	0.007*
Z-score of femoral neck aBMD^b^	−0.511 ± 0.828	−0.091 ± 1.056	0.016*
Bone morphometry			
Total area (mm^2^)^b^	182.5 ± 30.2	184.4 ± 25.6	0.707
Cortical area (mm^2^)^a^	19 (11.9–28.2)	26.8 (18.5–35.1)	0.003*
Trabecular area (mm^2^)^b^	150.3 ± 32.6	148.58 ± 26.41	0.750
vBMD			
Total vBMD (mg HA/cm^3^)^a^	240.7 (203.9–270.3)	259.8 (221.9–301.3)	0.088
Cortical vBMD (mg HA/cm^3^)^b^	654.5 ± 85	699.9 ± 74.7	0.002*
Trabecular vBMD (mg HA/cm^3^)^a^	147.4 (133.1–164)	147.5 (123.1–164.8)	0.575
Bone mechanical properties			
Stiffness (N/mm)^a^	41,886 (36,226.3–52,529)	48,240.5 (40,931–57,711.5)	0.005*
Estimated failure load (N)^a^	1,794.2 (1,504.2–2,123.8)	2,050 (1,774.3–2,364.2)	0.004*

^a^ Mann–Whitney *U* test was used on data which were not normally distributed. Median and range.

^b^ Student’s *t*-test was used on data with normal distribution. Mean and standard deviation.

^c^ Chi-square test was used for the dichotomous data.

* *p* ≤ 0.05.

**Figure 1:**
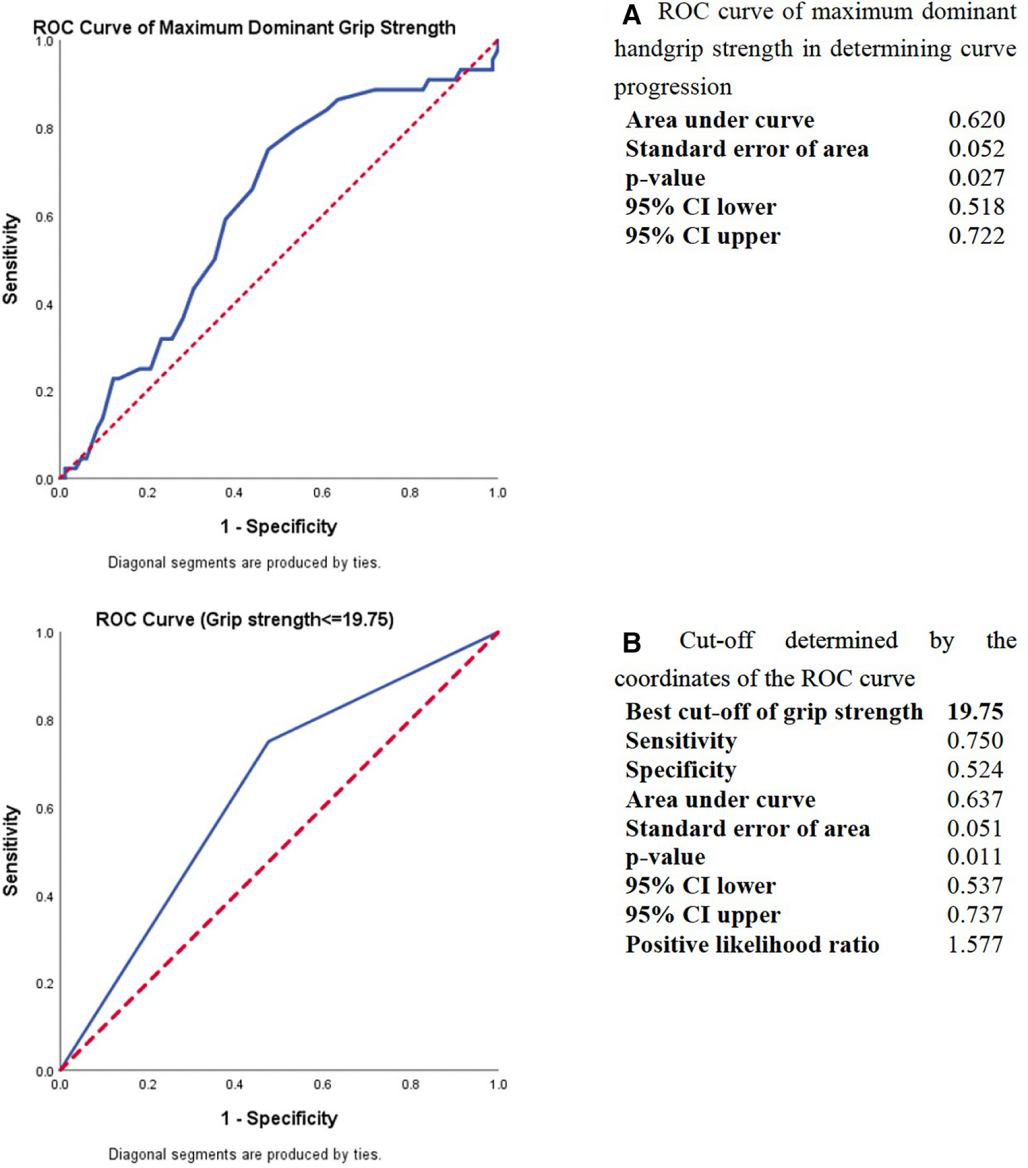
ROC curve analysis of maximum dominant handgrip strength in determining the curve progression in AIS. (**A**) ROC curve of maximum dominant handgrip strength in determining curve progression. (**B**) Cut-off determined by the coordinates of the ROC curve.

The predictive power of dominant handgrip strength in the curve progression was estimated using receiver operating characteristic (ROC) analysis. The best cut-off threshold of maximum dominant handgrip strength was 19.75 kg for distinguishing progressive AIS from stable AIS, with a sensitivity of 75.0%, specificity of 52.4%, and area under curve of 0.637 (Figure 1). When applying dichotomous result of maximum dominant handgrip strength into a backward logistic regression with all the significant predictors, the handgrip strength and estimated failure load were remained in the last step of the logistic regression (odds ratio = 2.325 and 0.999 with *p* = 0.080 and 0.062, respectively, Table 2).

**Table 2:** Backward logistic regression to predict the curve progressions based on the significant parameters in univariate comparison shown in Table 1.

	*B*	SE	*p*-value	exp(*B*)	95% CI for exp(*B*)	
					Lower	Upper
First step						
Weight	−0.065	0.073	0.374	0.937	0.813	1.081
TOCI	0.097	0.275	0.725	1.101	0.643	1.886
Trunk lean mass	−0.638	0.774	0.409	0.528	0.116	2.406
Non-dominant arm lean mass	−1.620	5.704	0.776	0.198	0.000	1.42 × 10^4^
Dominant arm lean mass	6.766	6.980	0.332	867.990	0.001	7.59 × 10^8^
Grip strength ≤19.75	0.915	0.548	0.095	2.497	0.853	7.309
Femoral neck aBMD	0.724	3.920	0.853	2.062	0.001	4,477.209
Cortical area	0.013	0.079	0.871	1.013	0.867	1.183
Cortical vBMD	−0.006	0.011	0.559	0.994	0.973	1.015
Stiffness	1.50 × 10^−4^	1.93 × 10^−4^	0.435	1.000	1.000	1.001
Estimated Failure Load	−0.004	0.005	0.422	0.996	0.985	1.006
Constant	7.844	7.414	0.290	2,549.352		
Nagelkerke *R*^2^	0.193					
Last step						
Grip strength ≤9.75^a^	0.844	0.482	0.080	2.325	0.904	5.981
Estimated failure load^a^	−0.001	0.001	0.062	0.999	0.998	1.000
Constant	0.933	1.305	0.475	2.542		
Nagelkerke *R*^2^	0.146					

B, coefficient; SE, standard error of *B*; exp(*B*), estimated odds ratio; 95% CI for exp(*B*), confidence interval for exp(*B*).

^a^ Handgrip muscle strength and estimated failure load were presented in the last step of the logistic regression (odds ratio = 2.325 and 0.999 with *p* = 0.080 and 0.062, respectively).

## 5. Discussion

This study represents the first scientific investigation on the association between bone qualities (bone size, vBMD, and bone mechanical properties) and muscle parameters (skeletal muscle mass, lean mass, and handgrip strength) among female patients with AIS. In addition, this research explores the potential application of baseline handgrip strength as a predictive parameter, in addition to existing bone parameters, in predicting curve progression in AIS. The results of this study showed that individuals with progressive AIS exhibited poorer arm lean mass and estimated trunk lean mass, weaker dominant handgrip strength, deranged cortical bone qualities, and lower bone mechanical properties at the baseline when compared with those with stable AIS. This study further showed that a cut-off threshold of 19.75 kg in the dominant handgrip strength, in addition to existing bone parameters, could potentially be used clinically as a predictive parameter in predicting curve progression in AIS.

Patients with AIS are found to have low bone mass (7), deranged bone qualities (5), and low bone mechanical properties (4) when compared with normal age-matched females. Several groups had previously identified some objective bone parameters in predicting curve progression. Hung et al. (7) reported that low bone mass at the femoral neck measured by DXA could predict curve progression in AIS. Yip et al. (8) identified that cortical vBMD at the distal radius measured by HR-pQCT is an important prognostic factor of curve progression to surgical thresholds. Individuals with AIS are known to have low lean mass (11) and muscle imbalance (15). A longitudinal study conducted in the United Kingdom reported that AIS patients who had lower lean mass at 10 years old would have higher risk of scoliosis at the age of 15 (34). Although previous studies have utilized bone parameters such as DXA and HR-pQCT to examine bone qualities, these measurements are often costly, not widely available in clinical settings, and require expertise for interpretation.

Muscle parameter measurements included in this study such as handgrip strength can be measured by a standard dynamometer, which is an easy, quick, and portable clinical assessment tool at a low cost. Handgrip strength is associated with the muscle strength in other areas of the body, including the erector of spine (35), shoulder abductors, and total muscle strength. Consequently, handgrip strength can be used as a rapid indicator of general muscle strength in adolescents (36). A recent large cohort study found that reduced handgrip strength is associated with lower BMD and BMC and concluded that handgrip strength can be a possible indicator of bone health in adolescent students (37). The present findings indicated that progressive AIS patients had both lower handgrip strength and lower bone qualities. The handgrip strength combined with estimated failure load measured by FEA was the best model in predicting curve progression in AIS. We further identified a cut-off threshold of 19.75 kg in this cohort for predicting curve progression. This cut-off threshold of 19.75 kg is close to the normative handgrip strength value of 19.8 kg in Hong Kong girls at 14 years old (38) and 20 kg in Korean girls between the age of 10 and 14 (39). It is slightly higher than the recommended handgrip strength cut-off threshold of 18 kg for sarcopenia diagnosis among older Asian female population (40). We speculated that AIS patients with sarcopenia-like low muscle mass in the trunk and weak muscle strength might have higher risk of curve progression.

With the advancement of genetic analysis, genome-wide association studies (GWAS) have identified several gene loci such as ladybird homeobox 1 (LBX1) (41, 42), basonuclin 2 (BNC2) (43), and fibrillin (FBN1 and FBN2) (44, 45), which are associated with the pathogenesis of AIS. Both LBX1 and BNC2 are functionally related to early muscle development (46), whereas fibrillins are components of connective tissue (47). Some hormones including melatonin, leptin, and calmodulin, which are related to muscle strength and contractility (48–50), are also observed to be connected to muscle deficits in individuals with AIS. Lower free leptin bioavailability was found in female patients with AIS with strong correlation to lower muscle mass and body fat (49). Reduced calmodulin was observed in patients with AIS receiving brace treatment and spinal fusion (51). The paraspinal muscles of AIS patients displayed an asymmetrical distribution of calmodulin and melatonin levels (50, 52). The findings from these studies provided evidence that the onset of scoliosis might be initiated by primary soft tissue and muscle anomalies to be accompanied with subsequent bone–muscle interaction (46) during curve progression, thus supporting the potential prognostic value of muscle parameters for patients with AIS.

The school screening program for AIS in Hong Kong was found to have higher positive predictive value (PPV) when compared with similar programs in other Asian countries (53). The PPV ranged from 35.6%–43.6% for curve angle of ≥20° and 4.3%–6.1% for curve angle of ≥40° (54). However, there is a need for more effective prognostication of significant curve progression during the initial clinical consultation in order to provide timely and necessary treatments. Due to the multifactorial nature of AIS, a recent study had proposed a prognostic composite model consisting of clinical and circulating parameters to predict the risk of curve progression to a severe Cobb angle of >40° with a sensitivity of 72.7% and a specificity of 90% (55). While the circulating parameters could only be obtained through invasive blood sampling in a clinical setting, a simple clinical assessment of handgrip strength, along with other clinical measurements including the forward bending test, angle of trunk rotation, and moiré photograph, can be easily performed in school screening program for early screening and identification of individuals with AIS who are at a higher risk of curve progression. In addition to the clinical parameters of anthropometry, maturity, and circulating parameters, the inclusion of handgrip strength assessment could be incorporated into existing prognostication models with curve factors (such as initial Cobb angle, curve type), patient factors (such as gender, age, and maturity), and bone factors (bone health status) to improve the predictive power of curve progression.

The relationship between the muscle and bone in AIS has not been fully investigated. Previous studies revealed the close interaction between the muscle and bone in which sarcopenia and osteoporosis share common mechanisms and risk factors (56, 57). The findings of the present study suggested that handgrip strength plays a more important role in predicting bone qualities and curve progression in AIS. According to the mechanostat hypothesis, muscle contraction provides mechanical stimulation to promote bone formation during growth (58). The bone in AIS might have a unique response to mechanical stimuli, which can be more prominent among progressive AIS. The poorer muscle health in individuals with AIS, particularly in those with progressive AIS, could be reflected by lower handgrip strength. Enhancing overall muscle strength by increasing physical activity could provide beneficial effects on cortical bone growth, including the thickening of the cortical bone and increasing cortical bone density.

There are several limitations in this study. First, the current study only included female subjects with a narrow age range in one single center. The relatively small sample size has limited the predictive ability of this univariate model, and there is undoubtedly room for enhancing performance. However, the current findings are useful to test our hypothesis on using muscle parameters such as handgrip strength to identify patients with higher risk of curve progression which may be indicative to inform timely management of AIS, particularly at the early stages of diagnosis, for better disease control. Second, data of physical activity and dietary calcium intake were self-reported by the subjects with the help from their guardians. Third, only muscle mass and strength were examined whereas other muscle parameters such as muscle size and composition were not included in this study. Fourth, we were unable to account for the changes in lifestyle between the baseline and skeletal maturity stages, as well as the impact of treatments, such as bracing, that were administered to our subjects throughout the duration of the study. The biochemical interaction between the muscle and bone was not studied as blood tests were not performed in the present study. The current findings would provide valuable insights for determining the appropriate sample size for future validation study assessing the translation and adaptation process. Further studies are warranted to include a larger sample size with a wider age range to enhance the predictive power and provide reference values for handgrip strength that are particular to age, ethnicity, and gender. It is also worthwhile to further explore various independent factors, such as handgrip strength assessment identified in this study and formulate these factors into the existing composite model to enhance the predictive sensitivity and specificity of this multifactorial and complex condition. The biochemical cross-talk between the muscle and bone with blood tests can also be investigated in order to unravel the whole picture of muscle and bone interaction in AIS.

In essence, progressive AIS is associated with lower handgrip strength and poorer bone mechanical property, which are important predictors of curve progression in AIS. In addition to existing bone parameters, the implementation of handgrip strength, which is a quick, portable, and low-cost assessment with a cut-off threshold of 19.75 kg in the dominant hand, could be useful for predicting AIS with higher risk of curve progression in order to facilitate timely management at an early stage of diagnosis.

## Data Availability

The datasets presented in this article are not readily available because of patient privacy and confidentiality. Requests to access the datasets should be directed to the corresponding author.
